# Probing Retroviral and Retrotransposon Genome Structures: The “SHAPE” of Things to Come

**DOI:** 10.1155/2012/530754

**Published:** 2012-05-17

**Authors:** Joanna Sztuba-Solinska, Stuart F. J. Le Grice

**Affiliations:** RT Biochemistry Section, HIV Drug Resistance Program, National Cancer Institute, Fredrick, MD 21702-1201, USA

## Abstract

Understanding the nuances of RNA structure as they pertain to biological function remains a formidable challenge for retrovirus research and development of RNA-based therapeutics, an area of particular importance with respect to combating HIV infection. Although a variety of chemical and enzymatic RNA probing techniques have been successfully employed for more than 30 years, they primarily interrogate small (100–500 nt) RNAs that have been removed from their biological context, potentially eliminating long-range tertiary interactions (such as kissing loops and pseudoknots) that may play a critical regulatory role. Selective 2′ hydroxyl acylation analyzed by primer extension (SHAPE), pioneered recently by Merino and colleagues, represents a facile, user-friendly technology capable of interrogating RNA structure with a single reagent and, combined with automated capillary electrophoresis, can analyze an entire 10,000-nucleotide RNA genome in a matter of weeks. Despite these obvious advantages, SHAPE essentially provides a nucleotide “connectivity map,” conversion of which into a 3-D structure requires a variety of complementary approaches. This paper summarizes contributions from SHAPE towards our understanding of the structure of retroviral genomes, modifications to which technology that have been developed to address some of its limitations, and future challenges.

## 1. Introduction

*Cis-*acting sequences within the (+) strand RNA genomes of retroviruses and long terminal repeat (LTR) containing retrotransposons control several critical events in their life cycle, including transcription [1], translation [2], dimerization [3], packaging [4], RNA export [5], and DNA synthesis [6]. Development of novel RNA-based strategies to ameliorate human immunodeficiency virus (HIV) pathogenesis would therefore benefit from an improved understanding of RNA structure and how this mediates interactions with both host and viral proteins. Historically, deciphering higher-order RNA structure has taken advantage of base- and structure-specific nucleases (e.g., RNases A, T1, T2 [7] and nuclease S1 [8]) or chemicals (e.g., dimethyl sulfate, diethyl pyrocarbonate [9, 10], and Pb^2+^ [11]). While these approaches have produced seminal advances in elucidating features of the HIV-1 and HIV-2 genomes [12–23], the necessity in most cases for multiple reaction conditions can be considered a limitation. Moreover, in almost all instances, enzymatic and chemical RNA footprinting has been performed on short RNAs prepared by *in vitro *transcription and labeled with ^32^P, eliminating any positional context, that is, regulatory roles that might be mediated by long-range, tertiary interactions. Although this challenge has in part been addressed by Paillart et al. via *ex vivo* footprinting of virion-associated RNA with dimethyl sulfate [24], a more “user-friendly" approach capable of providing information on RNA structure both *in vitro* and *ex virio, *and with fewer base-specific reagents, would clearly be advantageous.

 Selective 2′ hydroxyl acylation analyzed by primer extension (SHAPE), reported in 2005 by Merino and colleagues [25], has emerged as a facile technique that addresses many of these concerns. Since the target of the probing agent (N-methyl isatoic anhydride (NMIA) [25] or 1-methyl-7-nitroisatoic anhydride (1M7) [26]) is the ribose 2′ hydroxyl, all four RNA bases are simultaneously probed with a single reagent. Secondly, when combined with fluorimetric detection, multiplexing and automated capillary electrophoresis, SHAPE profiles of complete, 10,000 nt retroviral genomes can be generated in a matter of weeks [27]. By comparing reactivity profiles obtained *in vitro* and *ex vivo*, these studies have also provided important information on HIV genome organization and the role played by chaperone proteins. Finally, the recent advent of the self-inactivating electrophile benzoyl cyanide (BCN) [28] opens the possibility of time-resolved SHAPE, which promises to provide important glimpses into RNA conformational dynamics.

 Despite these benefits, it should be borne in mind that SHAPE effectively provides a secondary structure nucleotide “connectivity” profile; that is, it does not report directly on long-distance tertiary interactions such as kissing loops and pseudoknots and is best used in conjunction with other solution techniques, such as X-ray crystallography, NMR spectroscopy, and small angle X-ray scattering in order to generate an accurate 3-D model. Where possible, combining structural data with a genetic analysis, via construction of disruptive and complementary mutations, should be seen as an important complement. In this communication, we have reviewed the basic SHAPE methodology and its application to understanding the structure of regulatory elements of both retroviral and retrotransposon genomes. Modifications to the probing technology which have allowed us to (i) investigate tertiary interactions important for regulating nucleocytoplasmic RNA transport and (ii) combine chemical modification with tandem mass spectrometry to understand conformational dynamics of RNA/DNA hybrids containing polypurine tract (PPT) primers of (+) strand DNA synthesis, are presented. Finally, future challenges of SHAPE, including increasing sensitivity where the amount of biological material is limiting, and studying interconverting RNA structures, are also discussed.

## 2. SHAPE Methodology

**Figure 1:**
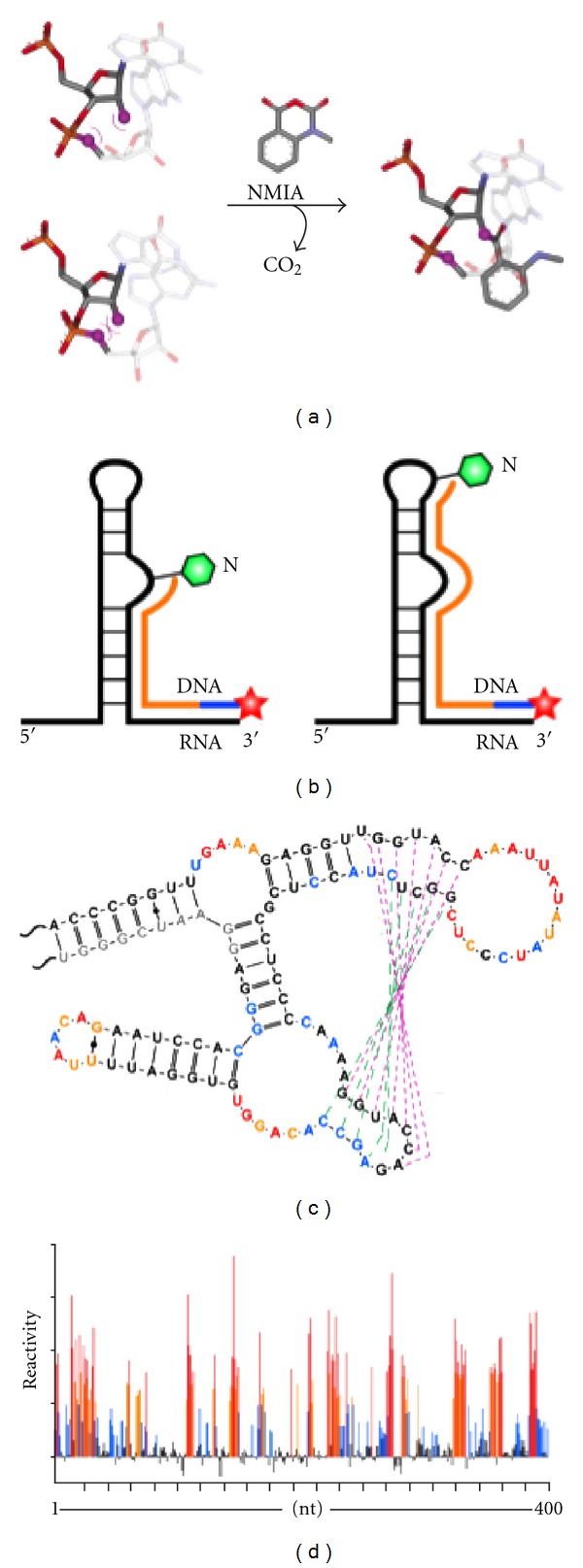
Overview of SHAPE technology. (a) Ribose 2′ OH of RNA at flexible, or unpaired nucleotides is selectively modified by NMIA. (b) Positions of adduct formation result in impaired primer extension during subsequent cDNA synthesis. (c) Radiolabeled or fluorescently-labeled primer extension products are resolved by high resolution polyacrylamide gel electrophoresis or automated capillary electrophoresis. (d) Electropherograms are computationally deconvoluted to obtain normalized NMIA reactivities, from which a secondary structure model is constructed.

A brief outline of SHAPE methodology is presented in Figure 1. As originally conceived, this chemoenzymatic strategy assesses local flexibility in RNA via accessibility of the ribose 2′-OH group to acylation by the electrophilic reagent NMIA. In flexible regions (such as loops, bulges, and junctions), RNA adopts conformations that will promote formation of a nucleophilic 2′-oxyanion which reacts with NMIA to form a bulky 2′-*O*-adduct [25] (Figure 1(a)). Recent modifications to the strategy have taken advantage of 1M7 [26] and BCN [28], which are more labile towards hydrolysis and self-inactivation, making them particularly advantageous for performing time-resolved footprinting. Modified RNAs are subsequently evaluated by primer extension with an RNase H-deficient reverse transcriptase, creating a cDNA library corresponding to stops at sites of adduct formation in the RNA when analyzed by high resolution gel electrophoresis (Figure 1(b)). End-labeling with ^32^P allows primer extension products of 50–300 nt to be fractionated by conventional denaturing polyacrylamide gel electrophoresis, while multiplexing with fluorescently-labeled primers and automated capillary electrophoresis permits resolution of 500–750 nt in a single electropherogram (Figure 1(c)). Finally, autoradiograms or electropherograms are quantified and computationally deconvoluted in order to obtain the energy-minimized RNA structure (Figure 1(d)).

 In contrast to the many benefits of SHAPE, analyzing sites of adduct formation by primer extension has limitations for structural studies aimed at very short RNAs. Since SHAPE information is tabulated indirectly through the length and frequency of a given cDNA, information on ~50  nt at the 3′ terminus of the RNA molecule is lost as a consequence of both primer binding and reduced processivity of the retroviral reverse transcriptase used for cDNA synthesis. In an attempt to address this shortcoming, Steen et al. [33] recently combined chemical acylation with sensitivity to exonucleolytic degradation, based on the observation that RNase R exonucleases processively cleave RNA in a 3′ → 5′direction. Screening several sources of RNases R identified an enzyme from *Mycoplasma genitalium* capable of processively degrading RNA, including through base-paired regions, but not beyond sites of adduct formation. The approach of RNase-directed SHAPE provides a facile and important complement to examine structural features at the termini of important regulatory RNAs. Although there is currently no commercial source for *Mycoplasma genitalium* RNase R, methods for purifying this enzyme from recombinant *E. coli *have been published [34].

## 3. SHA-MS Combines Chemical Acylation with Mass Spectrometry

**Figure 2:**
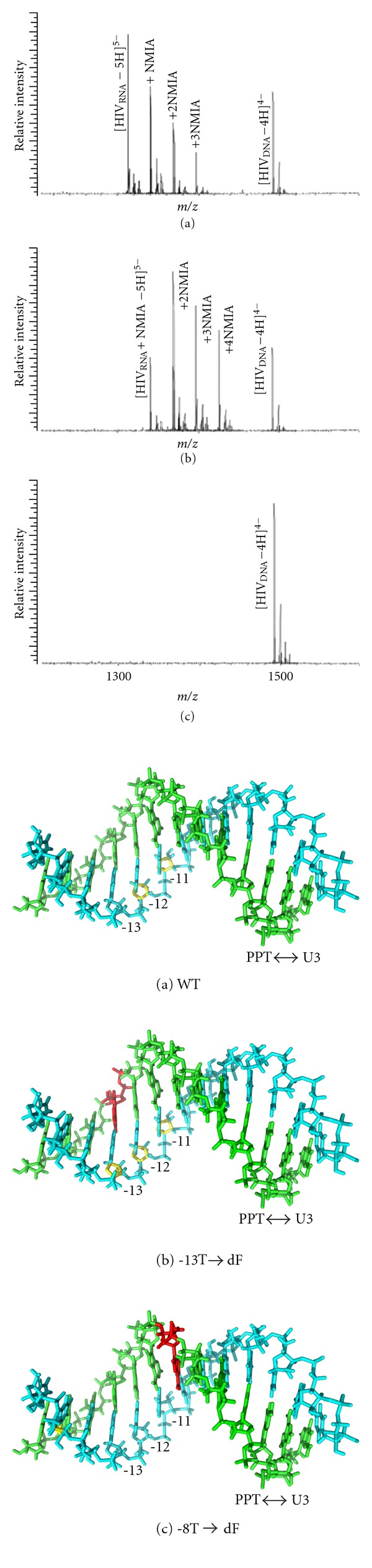
Examining RNA/DNA structural dynamics by combining chemical acylation with mass spectrometry. *Left*, Nano-ESI mass spectra of a model HIV-1 PPT RNA/DNA hybrid following treatment with a 10-fold (a), 50-fold (b), and 100-fold NMIA excess (c). At limiting NMIA concentrations (a) and (b), the majority of the PPT RNA is unmodified, and RNAs containing one, two, three, or four NMIA adducts can be observed, while excess acylation (c) results in overmodification of the entire RNA strand. In all cases, however, the PPT DNA complement is not modified by NMIA owing to the absence of a ribose 2′-OH group. *Right*, NMIA sensitivity of the wild type (a) and dF-modified (b) and (c) HIV-1 PPT RNA/DNA hybrids. In all cases, DNA and RNA nucleotides are represented in green and blue, respectively. NMIA-sensitive ribonucleotides are in yellow and positions of dF substitution in red. The position of the PPT/U3 junction has been indicated. Adapted from [29].

 As originally conceived, SHAPE was designed to interrogate structural features of RNA molecules ranging in size from several hundred to several thousand nucleotides. A critical feature of retrovirus and retrotransposon replication is initiation of (+) strand, DNA-dependent DNA synthesis from the polypurine tract (PPT) RNA primer. Although we have gleaned important information on PPT function using mutants of HIV RT [35–37] and targeted insertion of nucleoside analogs at, and in the vicinity of the PPT-U3 junction [38–42], the structural basis for PPT primer recognition remains elusive. Since our nucleoside analog strategy has mandated analysis of short RNA/DNA hybrids (25–30 bp), identifying structural anomalies by SHAPE becomes impractical. However, since RNA 2′-OH acylation results in a mass increment of 133 Da, we reasoned that adduct formation could be evaluated by electrospray ionization (ESI) mass spectrometry (MS). As illustrated in Figure 2(a), discrete PPT RNAs containing between one and four NMIA adducts could be detected by nanospray ESI-MS, while the DNA complement, as predicted, was insensitive to modification. Tandem mass spectrometry was subsequently used to define the positions of adduct formation indicating that, in addition to terminal ribonucleotides, which might be predicted to “fray,” ribonucleotides-11 and -12 of the wild type PPT (defining position -1 as the ribonucleotide 5′ of the PPT/U3 junction) were sensitive to acylation. These positions, corresponding to bases of the mispaired or “unzipped” component of the PPT observed crystallographically [43], suggest that either mispairing alters the geometry of the ribose 2′-OH or that the unzipped region of the PPT is transiently unpaired.

The utility of our approach [29], designated selective 2′ hydroxyl acylation analyzed by mass spectrometry (SHA-MS [29]), was perhaps better demonstrated by analyzing nucleoside analog-substituted PPTs. As might be predicted, substituting template thymine −13T with the nonhydrogen bonding pyrimidine isostere 2,4-difluorotoluene (dF [41]) expanded the NMIA sensitivity profile to include ribonucleotides-11, -12, and -13. However, replacing template nucleotide-8T with dF rendered not only primer nucleotides -11 and -12 insensitive to acylation, but also the complementary primer nucleotide -8, possibly indicating a local difference in base stacking that masks the ribose 2′-OH. Surprisingly, while the PPT RNA primer of the *Saccaromyces cerevisiae *LTR-retrotransposon Ty3 was insensitive to NMIA, acylation of ribonucleotide +1G was observed. These results were in agreement with NMR data [44], suggesting that a unique geometry at the Ty3 PPT/U3 junction may contribute towards recognition specificity. When complemented with KMnO_4_ footprinting, which differentiates between thymines in a single-stranded and duplex configuration [45], SHA-MS provides a valuable, high resolution approach to interrogate the geometry of short, purine-rich RNA/DNA hybrids where conventional probing strategies are impractical.

## 4. Antisense (AI)-Interfered SHAPE: Deciphering Tertiary Interactions

**Figure 3:**
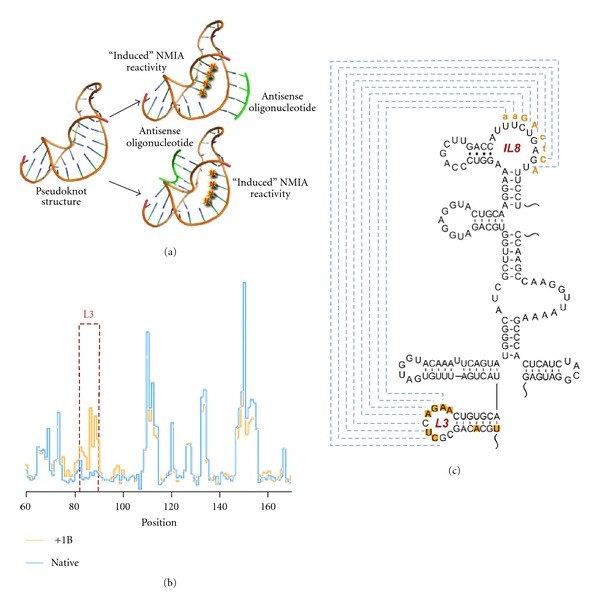
Examining RNA tertiary interactions by ai-SHAPE. (a) ai-SHAPE principal, that is, hybridization of an interfering oligonucleotide (green) to one partner of the proposed RNA duplex increases acylation sensitivity of its base-paired counterpart. (b) Electropherogram of NMIA reactivity of MTE nucleotides 60–170 in the absence (blue trace) and presence of the interfering oligonucleotide 1B (yellow trace). Loop L3 has been highlighted by the red box. (c) Secondary structure map for a portion of the *MusD* RNA transport element MTE, illustrating the L3/IL8 kissing interaction. The sequence of the interfering oligonucleotide hybridized to IL8 is indicated in orange, while nucleotides of loop L3 and the neighboring helix that exhibited enhanced NMIA reactivity are depicted within orange boxes adapted from [30].

Originally defined as an intermolecular interactions that mediate HIV-1 RNA genome dimerization [46], kissing loops have also been identified in the genomes of hepatitis C virus [47], chrysanthemum chlorotic mottle viroid [48], and a group C enterovirus [49]. Furthermore, pseudoknots, (tertiary interactions containing at least two stem-loop structures wherein a portion of one stem is intercalated between two halves of the other) are associated with translational control via internal ribosome entry sites [50], ribosomal frameshifting [51], and tRNA mimicry [52, 53]. Analysis of the RNA transport element of the murine retrotransposon *MusD* (MTE) revealed a complex structure containing a combination of a kissing loop and a pseudoknot [30]. Such tertiary interactions are particularly challenging for SHAPE and in the first instance require manual identification. In order to verify the identity of these structures, we developed an oligonucleotides-based interfering strategy designated antisense (ai)-interfered SHAPE, the basis of which is illustrated in Figure 3(a).

This strategy involves hybridization of short (5–10 nts) oligonucleotides to the proposed RNA duplex and determining whether this induces enhanced NMIA reactivity of the displaced strand. In view of their length, antisense oligonucleotides were constructed containing 2′-O-methyl and locked nucleic acid substitutions, both of which have been shown to improve duplex stability. Such interfering oligonucleotides are invasive inasmuch that they will hybridize to their partner sequence in an RNA that has already adopted its 3D structure. When applied to the MusD MTE, an interfering octanucleotide hybridized to internal loop 8 (IL8) stimulated NMIA reactivity at several positions in its kissing partner, loop 3 (L3, Figure 3(b)). Importantly, and as suggested earlier, the L3/IL8 kissing interaction suggested by ai-SHAPE was confirmed genetically *in vivo*, where MusD-dependent nucleocytoplasmic RNA transport was abrogated and restored by disruptive and compensatory kissing loop mutations, respectively. The structure of the MusD pseudoknot was likewise confirmed by ai-SHAPE, while a genetic analysis indicated that the ability to assume a pseudoknot configuration was a more critical determinant of function than absolute nucleotide sequence.

## 5. Interconverting RNAs: Choosing between Dimerization and Protein Synthesis

**Figure 4:**
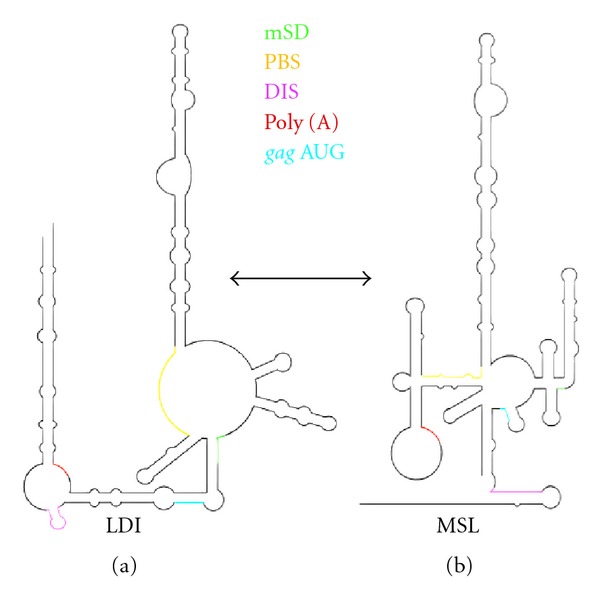
Proposed interconverting structures of the FIV 5′ leader RNA controlling genome dimerization/packaging and translation. For both the long-distance interaction (LDI) (a) and multiple stem-loops (MSL) structures (b), important regulatory sequences have been color-coded. mSD, major splice donor sequence; PBS, tRNA_Lys,3_ primer binding site; DIS, dimer initiation site; Poly(A), poly (A) hairpin; *gag *AUG, *gag* initiator methionine codon. See text for fuller details adapted from [31].

 Riboswitches, located in the noncoding region of several mRNAs, have been demonstrated to regulate RNA stability, protein synthesis, and splicing via a conformational change mediated by binding of a high-affinity ligand [54–56]. The highly-structured 5′ untranslated regions of many retroviruses can be considered formally analogous to a riboswitch, inasmuch as overlapping sequences have been proposed to mediate both genome dimerization/packaging and translation [57, 58]. An inconclusive acylation pattern in our recent SHAPE study of the 5′ UTR of the feline immunodeficiency virus (FIV) genome [31] led us to postulation that certain regions were metastable, allowing them to adopt alternative structures, a notion strengthened by the observation of two closely-migrating RNA species following fractionation by nondenaturing polyacrylamide gel electrophoresis. The hypothesis that best unified our experimental data is illustrated in Figure 4, suggesting that alternate structures for the FIV 5′ UTR mediate different events in the retrovirus life cycle.

 The long range interaction (LRI) model, originally proposed by Kenyon et al. [59], exposes the putative FIV dimer initiation sequence (DIS), while the *gag* initiation codon is embedded within a short helix, and free energy calculations suggest this model would support genome dimerization and packaging. The LRI structure also exposes the tRNA primer binding site from which reverse transcription is initiated following infection. The alternative, multiple stem-loop (MSL) structure occludes the DIS, while the *gag* initiation codon is positioned within a short stem-loop, the stability of which would facilitate translation over dimerization and packaging. In the MSL, the tRNA primer binding site is also inaccessible. Support for interconverting structures of the 5′ UTR was provided by the observation that the FIV mutant AN14, demonstrated *in vivo* to have impaired packaging [60], exhibited impaired dimerization *in vitro*, while dimerization was enhanced when the RNA was stabilized in the LRI form [31]. Though a later section will address future SHAPE strategies, our study of the FIV leader RNA provides another good example of combining chemical probing with functional studies, while at the same time highlighting one of its challenges, namely, how to deal with interconverting RNAs. One potential solution might be to perform nondenaturing electrophoretic separation immediately following chemical acylation. Since SHAPE relies on single-hit kinetics, modified RNAs should still resolve as discrete species. Polymerizing gels with disruptable crosslinking agents such as N,N^'^-bisacryloylcystamine (BAC) or N,N-diallyltartardiamide (DATD) would allow solubilization and recovery of nucleic acid for subsequent cDNA synthesis. Should ribose acylation alter RNA conformation, in-gel probing directly following fractionation by nondenaturing electrophoresis is an alternative strategy.

## 6. Investigating RNA Tertiary Structure with “Threading Intercalators”

**Figure 5:**
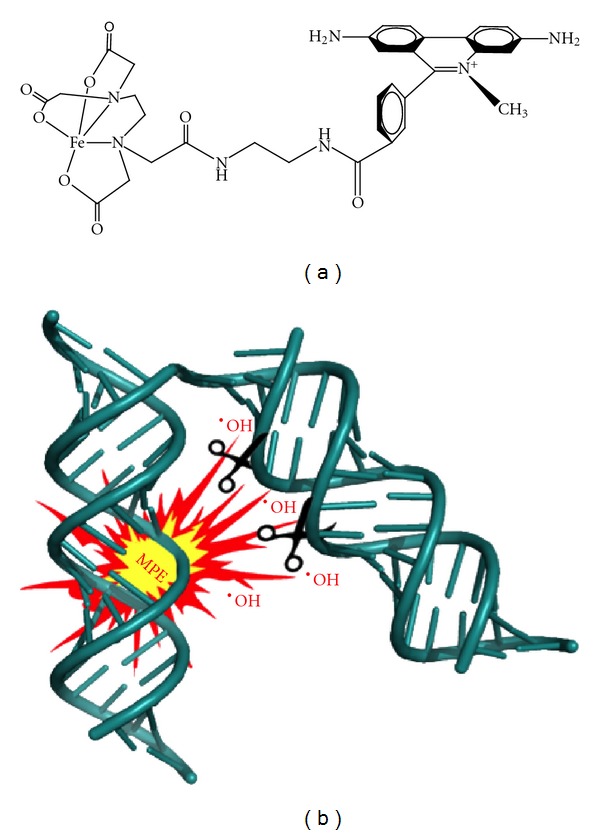
(a) Structure of the threading intercalator, MPE. (b) Examining RNA tertiary interactions by through-space hydroxyl radical cleavage (–OH) with the threading intercalator methidiumpropyl EDTA (MPE). Once a SHAPE profile for the RNA under investigation is determined, an MPE intercalation site is introduced by replacing four consecutive nucleotides with the CGAG/C(C/U)G recognition motif. SHAPE is then repeated to determine that sequence changes are nonperturbing, after which site-directed hydroxyl radical cleavage is performed to identify neighboring sites in the RNA. Repeating this process with independent RNAs containing unique MPE intercalation sites cumulatively provides information on tertiary interactions.

Understanding RNA structure-function relationship requires accurate three-dimensional structure modeling methods. At present, there is a substantial gap in obtaining high-throughput 3D information for RNA molecules larger than 150 nts. The techniques frequently used to obtain atomic resolution of RNAs, such as NMR spectroscopy and X-ray crystallography, have restrictions that preclude structural analysis. In NMR spectroscopy, the excited signal from individual atomic nuclei becomes congested and difficult to analyze with the increasing size of RNA molecule. Even though X-ray crystallography does not suffer from size limitations, obtaining crystals for flexible and diverse RNA structures represents a great challenge. These difficulties however, are now being addressed by combining SHAPE with methidiumpropyl-EDTA- (MPE-) directed through-space hydroxyl radical cleavage, as outlined schematically in Figure 5. In the past, MPE has been successfully applied as a tool for footprinting binding sites of small molecules on heterogeneous DNA [61], RNA folding analysis [61, 62] and examining RNA-binding properties of phospho- and dephospho-RNA-dependent protein kinase [63]. Recently, Gherghe et al. successfully combined SHAPE with MPE-directed hydroxyl radical cleavage to study tRNA^Asp^ tertiary structure [64].

MPE is a methidium intercalator moiety tethered to EDTA that preferentially intercalates at G-C rich helices in RNA at sites adjacent to a single nucleotide bulge. The intercalated MPE occupies roughly the same space as a single base pair and is oriented in the motif such that the EDTA moiety points toward the bulge. Upon addition of Fe(II) and a reducing agent, ferrous ion binds the EDTA and generates short-lived hydroxyl radicals that cleave proximal regions of the RNA backbone [65]. The MPE binding site can be placed at RNA helical motifs by replacing four consecutive base pairs with CGAG/C(C/U)G motif [64]. Provided that this replacement is compatible with the native structure of RNA, cleavage at positions proximal in space to the unique location of the bound MPE affords information about the nucleotides neighboring the intercalating ligand. Cleavage intensity at each position can be calculated as a ratio relative to the mean value for all intensities, after subtracting background cleavage observed for the native RNA sequence that does not contain an MPE binding site. Subsequently, MPE-directed through-space cleavage experiments yield high quality, long range constrains that refine nucleotide positions in RNA to atomic resolution of 4 Å rmsd [64]. As a result, the combined experimental and computational approach has the potential to yield native-like models for functionally crucial RNA molecules. Currently, MPE is not commercially available, and its application to through-space cleavage has only been demonstrated with a well-characterized RNA (yeast tRNA^Asp^). However, synthesis of MPE has been reported, and this strategy opens the intriguing possibility of developing “molecular rulers” by introducing linkers of different length between the intercalating and hydroxyl radical generating moieties.

## 7. Bringing It All Together-Determining Full Genome Structures by SHAPE

Most structural analyses have historically targeted small RNA motifs (<500 nt) in artificial contexts and, in the absence of complementary genetic and phylogenetic data, may not accurately relate their structures to the biology of the larger RNAs from which they were derived. In contrast, SHAPE provides an unprecedented opportunity to view an entire RNA molecule, giving the researcher the opportunity to connect simple elements to the components of larger RNA motifs. This concept has recently been exemplified through the application of SHAPE to decode the structure of the entire HIV-1 genome (~9750 nucleotides) at single-nucleotide resolution [27]. This seminal study determined that, although the HIV-1 genome is less structured than ribosomal RNA, it nonetheless contains independent RNA folding domains. Some functionally significant RNA motifs were shown to belong to the larger elements, an example of which is the *gag-pol* ribosomal frameshift signal, which constituted one component of a three-helix structure (P1-P2-P3). The slippery sequence forms one of the three helices (P2), while two others (P1 and P3) are stabilized by an anchoring stem with two bulges. Additional RNA elements were identified in protein-coding regions of the genome, from which it has been tentatively postulated that RNA structure constitutes an additional organizational level of the genetic code. Since many proteins appear to fold co-translationally, highly structured RNA might induce pausing of the translational machinery, promoting protein folding in a more native-like conformation. In contrast, highly unstructured regions were observed in hypervariable regions of the HIV-1 genome, which have important roles in viral host evasion. These unstructured regions were shown as separated from the rest of the genome by stable helices that have been proposed to function as structural “insulators.”

The versatility of SHAPE extends to studying viral RNA not only in the context of the intact genome, but also at different biological states, providing information with respect to RNA conformational changes underlying different stages of viral life cycle. As an example, Wilkinson et al. [66] have provided structural information on the HIV-1 leader RNA in four biological states, namely (i) *in vivo*, (ii) *ex vivo*, where genomic RNA had been gently deproteinized, (iii) *in vivo*, but where important interactions between the nucleocapsid protein (NC) and genomic RNA had been compromised by covalent modification with aldrithiol-2 (AT-2 [67]), and (iv) genomic RNA prepared by *in vitro* transcription. This study concluded that the first 1000 nt of the HIV-1 genome exists in a single, predominant conformation in all four states. RNA of noncoding regions that regulate different steps of viral life cycle was distinguished by significantly lower sensitivity to acylation (predictive of secondary structure) than coding regions. A comparison of acylation profiles for the *in vivo* state with those following covalent modification by AT-2 defined several high affinity NC recognition sites, consistent with the role of this critical RNA chaperone in governing packaging of viral RNA. All NC binding sites were characterized by a G-rich single-stranded sequence flanked by stable helices. Additionally, RNA motifs where NC increases local flexibility were also identified, comprising single-stranded A/U-rich motifs adjacent to a duplex in which the first base pair includes a guanosine nucleotide. Collectively, this genome-probing approach suggests that local protein interactions can be organized by the long-range architecture of RNA. Although a limited region of the genome of the formerly known gammaretrovirus xenotropic murine leukemia virus related virus (XMRV) was examined using this strategy, it yielded similar conclusions on high affinity NC binding sites [68]. Future studies directed towards whole-genome structural analysis would, however, benefit from development of methods that enhanced SHAPE sensitivity, thereby reducing the culture volumes of potentially biohazardous material required. Efforts in this direction are discussed in the following section.

## 8. Increasing SHAPE Sensitivity for *In Vivo *Structure Analysis

 In most instances, RNA structural analysis is performed on material either made synthetically or via *in vitro* transcription, where the amount of starting material is not a major consideration. Although *in vivo* and *ex vivo *analysis of the entire HIV-1 genome has been reported [27], this has required virus isolation from substantial culture volumes and is not readily adaptable to routine laboratory procedures. Thus, in circumstances where the amounts of biological material may be both biohazardous and limiting, methods of increasing SHAPE sensitivity that have broader applicability would be a major advantage. Efforts in this direction are summarized below.

### 8.1. (i) SHAPE-Seq

**Figure 6:**
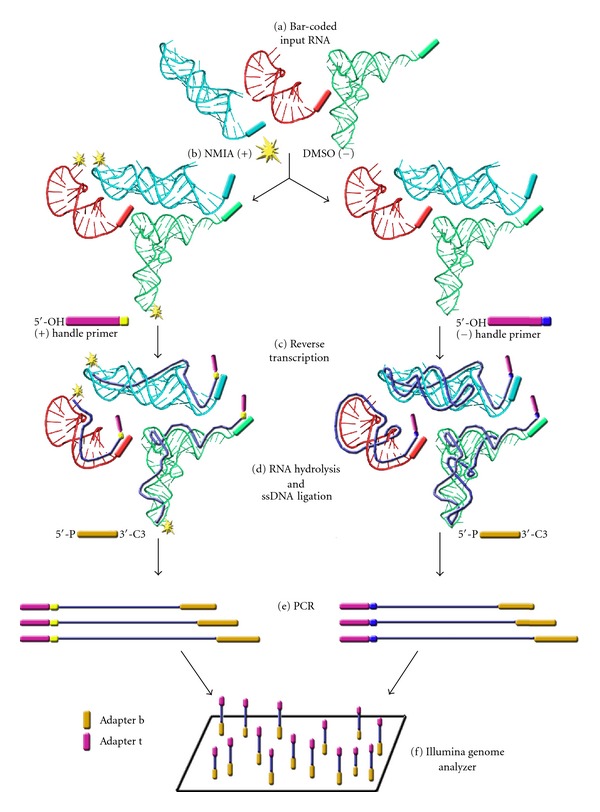
Summary of SHAPE-Seq methodology. (a) Input RNAs are bar-coded during *in vitro* transcription, followed by refolding under desired conditions and modification with SHAPE reagent (NMIA, 1M7). (b) The mixture is split into NMIA-treated and control pools. (c) Reverse transcription is performed with end-labeled primer containing a “handle” at the 5′ end and an Illumina adapter t. (d) The process is followed by hydrolysis of RNA and single-stranded (ss) cDNA ligation to incorporate the second Illumina adapter b. (e) After 9 to 12 cycles of PCR amplification, the cDNA library is analyzed by multiplex paired-end deep sequencing (f) adapted from [32].

Approximately 1–3 pmol of RNA is usually needed to accurately map a reactivity spectrum for any given RNA molecule [69]. This limits the application of SHAPE to biological samples for which significant amounts of RNA are available. The recently-described SHAPE-Seq technology provides a means of signal intensification to address this limitation [32]. This innovative methodology, which merges SHAPE with a multiplexed hierarchical bar coding and deep sequencing strategy, is outlined schematically in Figure 6.

Initially, input RNA templates are bar-coded with a unique sequence. Such barcodes comprise tetranucleotide sequences that are placed in the 3′ structural cassette and introduced prior to *in vitro* transcription. Subsequently, these RNA templates are mixed and refolded under desired conditions. After folding, the mixture is divided into two pools, one of which is treated with modifying agent, while the second treated with a control solvent. Primer extension is subsequently performed with an end-labeled DNA primer tagged at the 5′ end with tetranucleotide “handle” sequence. This handle allows the user to distinguish between cDNA fragments derived from the positive or control reactions. Additionally, the 5′ tail of the reverse transcription primer contains an Illumina adapter necessary for paired-end sequencing. As a result, reverse transcription generates a bar-coded library of uniquely-sized cDNAs corresponding to stops at sites of adduct formation in the target RNA. The process is followed by hydrolysis of RNA and single-stranded (ss) cDNA ligation to incorporate the second Illumina adapter. Single-stranded cDNA ligation is achieved using a thermostable ligase (circLigase, Epicentre Biotechnologies, Madison, WI) and a blocking group on the 3′ end of the adapter to prevent concatemerization [70]. Finally, 9 to 12 cycles of PCR, employing primers that bind to the Illumina adapter sequences, amplify the cDNA library before multiplex paired-end deep sequencing of primer extension products. Since the RNA modification position and the identity barcode are on opposite ends of the cDNA fragments, only 50 nucleotides need to be read on each terminus. After sequencing, the reads are separated first by handle sequence, then barcode, and subsequently aligned to probed RNAs.

When compared to conventional SHAPE, SHAPE-Seq permits rapid, fully-automated analysis and eliminates the necessity for manual, time-consuming data manipulations associated with quantification of fluorescently-labeled cDNAs by capillary electrophoresis. By ligating single-stranded cDNA products with 5′ adapters followed by PCR-amplification, with minute amounts of RNA needed to generate the reactivity spectrum of a given RNA, SHAPE-Seq represents a more generally-applicable and sensitive technique studying RNA samples that are limiting, from a biohazardous source, or both. For example, it was shown for the RNase P specificity domain that with as little as 0.1 pmol of input RNA, SHAPE-Seq reactivities of over 800 bar-coded RNA species could be inferred [32]. SHAPE-Seq has the additional advantage of being able to simultaneously determine structural information from many RNAs through direct sequencing of the 3′ RNA bar codes. Although the additional steps of SHAPE-Seq, (adapter ligation, PCR amplification, sequencing) might result in decreased sensitivity to some structural effects, as has been observed for the UUCG tetraloop of RNase P, this is offset with the ability of this technique to study structural changes involving interaction of various species within a population of RNA molecules.

### 8.2. (ii) Femtomole SHAPE

Using a two-color automated capillary electrophoresis with subfemtomole sensitivity, Grohman et al. [68] have recently reported *in vivo* analysis of a short portion of the formerly known XMRV genome. In contrast to earlier *in vivo *studies that required 1–3 pmole of input RNA, acylation profiles could be obtained with as little as 50 fmole aliquots of genomic RNA. As might be predicted, structural features of the XMRV leader RNA were similar to the extensively-studied counterpart Moloney murine leukemia virus, although binding sites unique to the XMRV nucleocapsid protein were proposed. More importantly, this study, which required in-house construction of a dedicated two-color capillary electrophoresis instrument, opens the exciting prospect of future functional studies on low abundance RNAs of clinical significance.

## 9. Future Perspectives

Rather than giving an exhaustive review of projects that have made use of SHAPE, which have included structures of wild type and mutant variants of the HIV-1 Rev response element [71], NC binding sites of the HIV-2 leader RNA [72], and RNA control of foamy virus protease activity [73], we have attempted here to highlight variations in this novel technology which facilitate interrogation of retroviral RNAs varying in size from 25–30 nt to intact, 9.5 kb retroviral genomes. The unequivocal benefit of this strategy is its ability to interrogate all four RNA bases with a single reagent, requiring thereafter simply fractionation of cDNA products. However, we should stress that SHAPE, while predictive of RNA structure, is best used with complementary genetic, phylogenetic, chemical modification (Pb^2+^ cleavage, ai-SHAPE and threading intercalators) and biophysical approaches (X-ray crystallography, NMR spectroscopy and small angle X-ray scattering). The benefits of capillary electrophoresis-based high throughput SHAPE must also be balanced by the demand this makers on the number of fluorescent oligonucleotide primers required for multiplexing, and the necessity for expensive instrumentation, features that also hold for femtomole SHAPE and SHAPE-Seq. Moreover, Kladwang and coworkers [74] compared SHAPE and crystallographic data for six RNAs and demonstrated significantly high (~20%) false negative and discovery rates, as well as several helix prediction errors, concluding that helix-by-helix confidence estimates may be critical for interpreting results from this powerful methodology. These issues notwithstanding, SHAPE should be seen as the beginning, and not the end, of an exciting path towards understanding the architecture of retroviral RNA genomes and the contribution this makes to biological function.
